# Multiparametric cloth-based wearable, SimpleSense, estimates blood pressure

**DOI:** 10.1038/s41598-022-17223-x

**Published:** 2022-07-29

**Authors:** Prashanth Shyam Kumar, Pratyush Rai, Mouli Ramasamy, Venkatesh K. Varadan, Vijay K. Varadan

**Affiliations:** Nanowear Inc., Brooklyn, NY USA

**Keywords:** Cardiology, Biomedical engineering, Statistics

## Abstract

Targeted maintenance of blood pressure for hypertensive patients requires accurate monitoring of blood pressure at home. Use of multiparametric vital signs ECG, heart sounds, and thoracic impedance for blood pressure estimation at home has not been reported previously. In an observational multi-site study, 120 subjects (female (N = 61, 52%)) between 18 and 83 years of age were recruited with the following stratification (Normal (20%), prehypertensive (37%), stage 1(26%), and stage 2 (18%). From these subjects, 1686 measurements of blood pressure from a sphygmomanometer were associated with simultaneously acquired signals from the SimpleSense device. An ensemble of tree-based models was trained with inputs as metrics derived from the multiparametric and patient demographics data. A test Mean Absolute Difference (MAD) of ± 6.38 mm of Hg and ± 5.10 mm of Hg were obtained for systolic and diastolic blood pressures (SBP; DBP), respectively. Comparatively, the MAD for wrist-worn blood pressure cuff OMRON BP6350 (GUDID—10073796266353) was ± 8.92 mm of Hg and ± 6.86 mm of Hg, respectively. Machine learning models trained to use multiparametric data can monitor SBP and DBP without the need for calibration, and with accuracy levels comparable to at-home cuff-based blood pressure monitors.

## Introduction

Hypertension is a critical public-health challenge worldwide. Between 1990 and 2019, the number of men and women with hypertension doubled to 652 million and 626 million. A pooled analysis of 1201 population-representative studies suggested that a dual approach of reducing hypertension prevalence through primary prevention, treatment, and control is achievable in the full spectrum of income settings^1^. Recently, Zhang et al. demonstrated the significance of intensive blood-pressure control in older Chinese patients resulting in a lower incidence of cardiovascular events^2^.

The potential sources of errors in blood pressure measurement remain a significant challenge even in controlled clinical settings. Discrepancies in measurements as obtained by different operators raise the concern of methodologic errors by one operator, such as under-cuffing, excessive pressure on the head of the stethoscope, rapid deflation of the cuff, or use of different arms. Kallioinen et al. investigated 29 potential sources of inaccuracy and categorized them as relating to the patient, device, procedure, or observer. They found significant directional effects with 27 sources with some inconsistency in terms of the direction of whether increase or decrease in measured BP. Some of the significant sources of directional effects caused changes in the range of  − 23.6 to + 33 mmHg SBP and − 14 to + 23 mmHg DBP^3^. Apart from the inaccuracies of measurements leading to incorrect hypertensive classifications, psychophysiological changes in patients with conditions such as white coat hypertension can lead to a false positive diagnosis of hypertension. The recommended measurement of BP for the diagnosis of hypertension across several international guidelines is out-of-office or at-home BP measurement using ambulatory BP monitoring (ABPM) and Home BP monitoring (HBMP). The available evidence suggests that HBPM and ABPM present similar values and correlate with target-organ damage^4^. However, there are challenges with both HBPM and ABPM.

Regarding HBPM, although patients are trained on how to perform an at-home BP self-measurement, the circumstances that must be created for repeatability of measurements are impractical for some patients, and compliance to these instructions is also not guaranteed. In the case of ABPM, the operation of the cuff during a measurement is perceptible to the patient and makes the measurements susceptible to a condition like white coat hypertension. Some patients must perform physical work such as construction that precludes the use of an ambulatory monitor, which requires the cessation of all movements during the oscillometric measurement time window. Furthermore, cuff-based ABMP is known to interfere with sleep^5^. Reports on sleep quality while using a cuffless blood pressure monitoring device (CLBPM) that is based on pulse wave analysis from a finger photoplethysmography sensor are scarce. The sleep quality was found to be comparable to ABPM^6^.

All commercially available ABPM and HBPM devices are calibrated to measure brachial BP, not central BP. A systematic review of invasive validation studies on the accuracy of estimation of aortic SBP using non-invasive devices could not draw specific conclusions^7^. Clinically, such devices are not yet prevalent due to significant variability in the estimation of aortic-SBP, and the superior invasive measurement methods are only indicated for patients suspected of having coronary artery disease^8^. The clinical evidence only supports a marginal difference. It is plausible that the incremental value of using central instead of brachial BP is masked by the errors in measurement of ABPM and HBPM, which are not encountered with invasive central BP measurements. Therefore, marginal superiority central BP offers as a risk predictor of cardiovascular events may be accessible with brachial BP by improving the calibration method of the brachial or peripheral BP monitoring device^8^.

**Figure 1:**
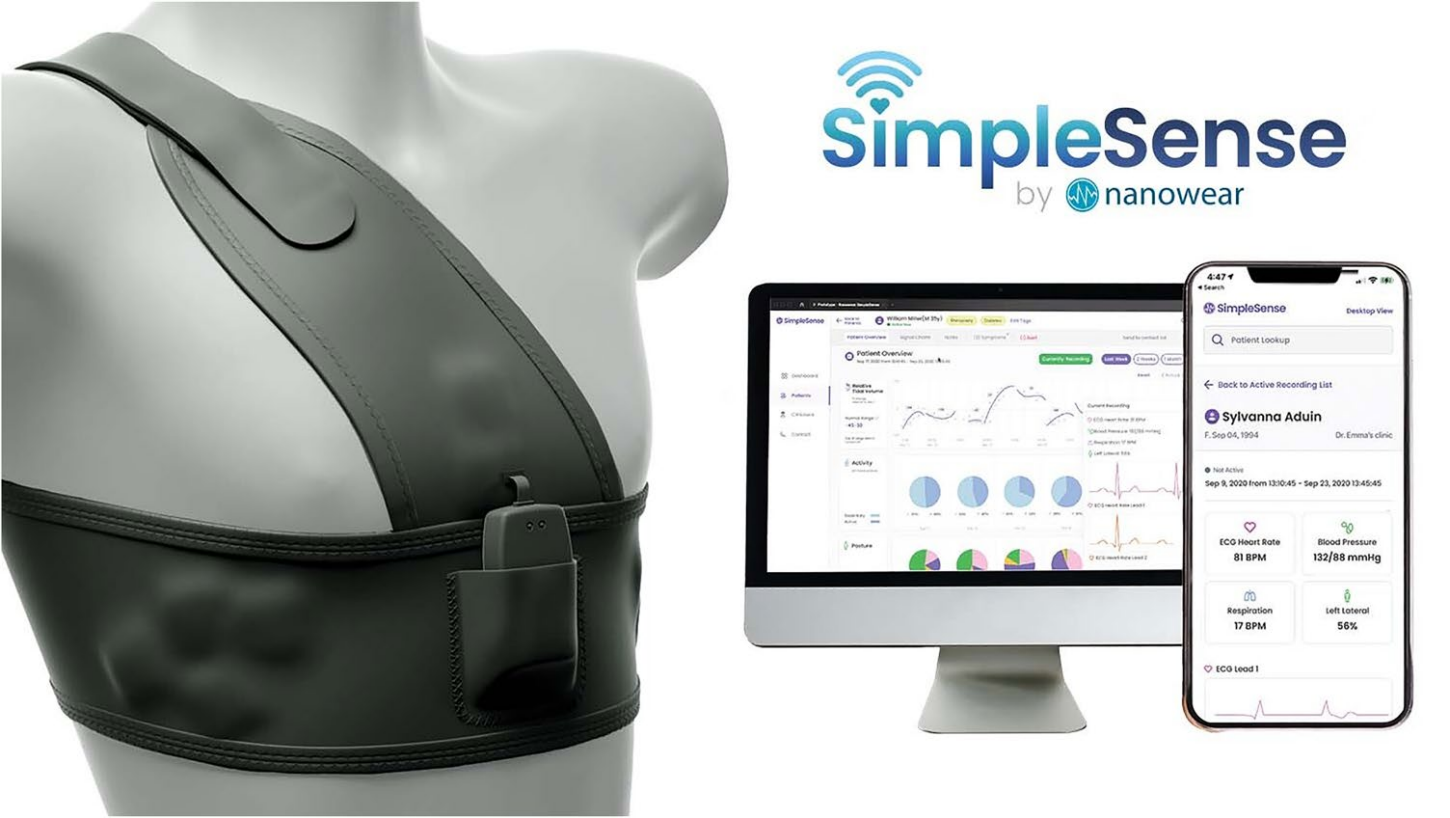
SimpleSense device and software platform.

With the advent of automated CLBPM, observer-related sources of errors may be mitigated by eliminating the subjective intra- and inter-observer variance. Device-related errors cannot be eliminated with any of the reported CLBPM methods that use the pulse waveform characteristics such as Pulse Wave Decomposition Analysis^9^, Pulse Wave Transit Time^10^, Pulse Arrival Time^11^, and estimation of the pre-ejection period because these methods require calibration against the readings from a cuff based sphygmomanometer for each patient. There are known confounders that decorrelate the relationship between photoplethysmography (PPG)-based parameters and SBP and DBP, such as changes in arterial stiffness (which increases with age as Elastin in the arteries is gradually replaced by less elastic Collagen), arterial wall viscosity, and assumptions on the radius of the arterial lumen (gender and BMI-related difference). None of the methods reported in the literature thus far have considered these demographic data to develop a generalized cuffless BP estimation method. Studies that have reported the use of only centrally measured cardiovascular parameters for the prediction of BP are scarce. Innovative use of ECG and deep learning to predict BP was reported^12^ but did not meet the minimum criteria for accuracy as per the IEEE and ANSI standards. In this paper, a novel approach is presented to estimate SBP and DBP that uses only centrally measured physiological parameters. The method takes as input the multiparametric data inclusive of two channels of ECG and thoracic impedance, heart sounds near the apex of the heart, activity, and posture captured by the FDA cleared SimpleSense device (510 (k) number K212160) (Nanowear Inc. Brooklyn, NY) combined with demographics data (age, gender, height, and weight) which is added as a predictor so that the confounding effects encountered by pulse wave-based techniques could be potentially mitigated. SimpleSense is a non-invasive, wearable, and portable medical device that uses cloth-based nanosensor technology^13^ (Fig. 1). The garment was designed with an emphasis on ease of wearing and takes between 20 and 30 s for most subjects to put on. Finally, two ensemble regression tree models trained using data from 120 subjects are used to estimate SBP and DBP. The accuracies of these models are presented.

## Methods

### Study design

A prospective multicenter non-randomized observational study was performed at Nanowear Inc. R&D in State College, PA, and ClinCept LLC, Atlanta, GA. The study consisted of executing a protocol with each subject involving activities that modulate the blood pressure for a duration of up to 1 h and 30 min. Nanowear Inc sponsored the trial. The study protocol (IRB protocol # 14-90-0093) was approved by the independent Institutional Review Board (IRB) Advarra Inc. (Columbia, MD). The study was conducted according to the stipulations of the 1964 Declaration of Helsinki and in accordance with relevant FDA regulations. Written informed consent was sought and obtained from all subjects and all subjects completed the study. The accuracy of the estimated blood pressure was evaluated following a validation plan based on the IEEE standard 1708a-2019^14^.

### Patient population

From February 8 to July 16, 2021, subjects were screened for eligibility and recruited. The targeted subject selection and stratification are presented in Table 1. The subjects were sorted into four cohorts—normal, prehypertensive, Stage 1 hypertension, and Stage 2 hypertension- based on the specified range of BP in Table 1. The endpoint of the BP classification was determined according to the JNC 7 report^15^. The BP used for classification was the entry BP measured at the beginning of the test. Three measurements were taken from the subject sitting with the elbow, back of the elbow, and back resting on a chair. The averaged value was used as the entry BP to determine the subject's BP classification.

**Table 1:** Subject selection requirements.

Blood pressure classification	Systolic blood pressure (mmHg)		Diastolic blood pressure (mmHg)	Subjects in Dev. set	Subjects in the test set
Normal	< 120	and	< 80	5	≥ 16
Prehypertension	120–139	or	80–89	5	≥ 16
Stage 1 hypertension	140–160	or	90–100	5	≥ 16
Stage 2 hypertension	≥ 160	or	≥ 100	5	≥ 16
Gender: At least 26 males and 26 females					

### Study procedure

Two trained observers performed all measurements using the SimpleSense device and gold standard sphygmomanometer for each subject. The training of the observers was as described in Section 5.2.2 of ISO 81060-2019.

### Blood pressure measurement

First, the subjects wore the SimpleSense system. The device recorded data while the two observers took the blood pressure measurements using a standard sphygmomanometer and Omron blood pressure wrist cuff GUDID 10073796266353. The simultaneously and synchronously acquired data from SimpleSense was used to calibrate the algorithm against the blood pressure measurements from the sphygmomanometer.

**Figure 2:**
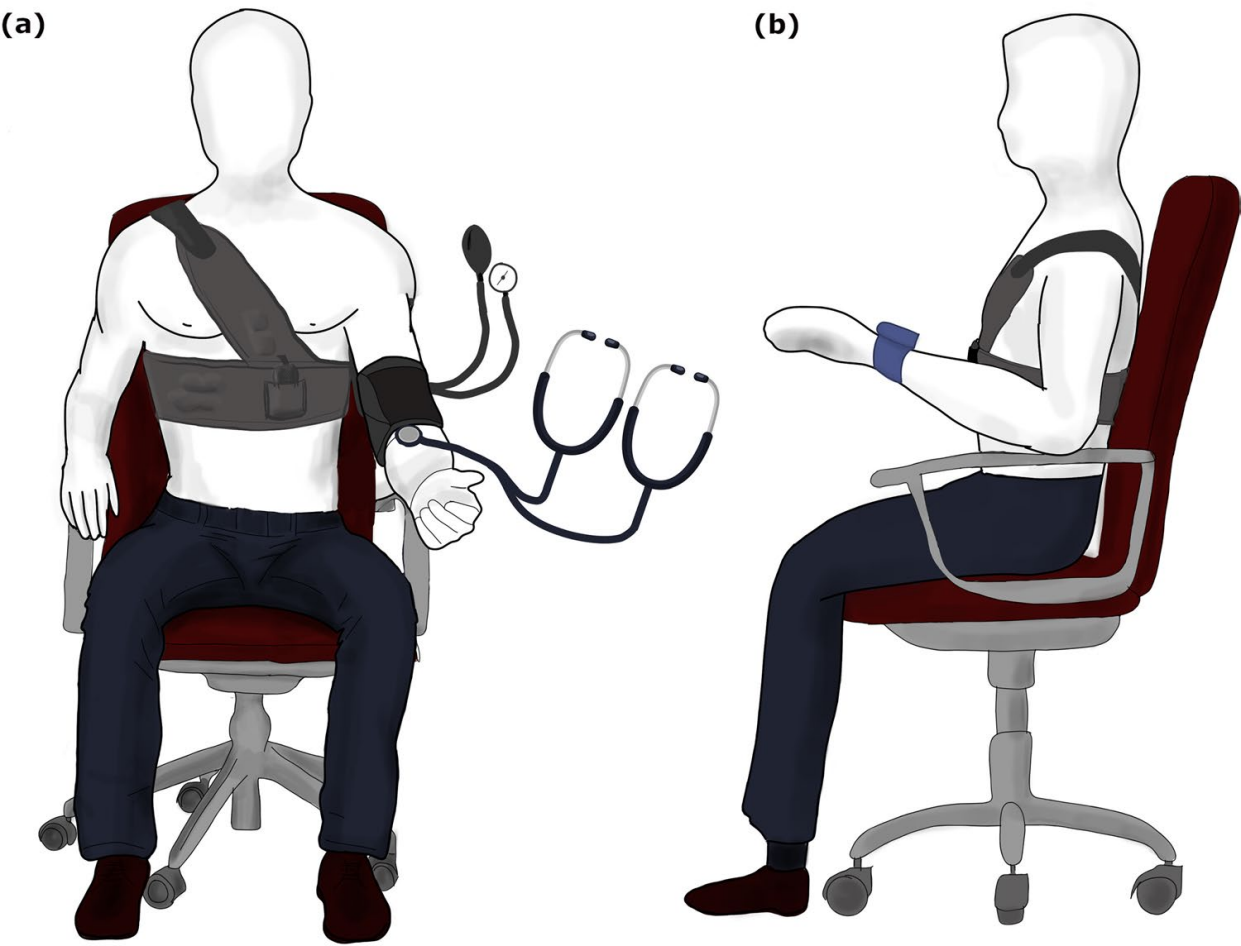
(**a**) Test set up for simultaneous SimpleSense recording and BP readings from Sphygmomanometer (**b**) Placement of Omron Wrist cuff and SimpleSense for sequential measurement.

Reference measurements were provided by the two trained observers and measured simultaneously with one reference sphygmomanometer (using a "Y" connector), as shown in Fig. 2a. The sphygmomanometer used as the reference standard met the requirement in ISO 81060-2:2019 or ANSI/AAMI SP10. SBP and DBP measurements with the sphygmomanometer were determined using the Phase 1 and Phase 5 Korotkoff sounds, respectively. The observers set up the sphygmomanometer and Omron wrist cuff, as shown in Fig. 2b. The wrist cuff and the sphygmomanometer were worn on the same (left) arm. All measurements were recorded to the nearest two mmHg. If both measurements from the two observers are no more than four mmHg apart, the mean value of the two was used as the reference measurement. Otherwise, the measurement was retaken. If either observer detected significantly irregular heart rhythm, that reading was excluded. The time of blood pressure measurement was recorded accurately to the nearest minute. The supplementary material includes the detailed test procedure.

Simultaneous measurement was used for the sphygmomanometer and SimpleSense device. The left arm was used for reference measurement. By design, the SimpleSense system is a cuffless device and does not rely on an arm or wrist cuff for blood pressure measurement. It can record all the physiological parameters during the inflation and deflation of the reference sphygmomanometer without any mutual interference. At least a 60-s delay was observed between consecutive readings from the sphygmomanometer to avoid venous congestion.

Sequential measurement was used for the Omron Wrist Cuff. When the wrist cuff device is used with the sphygmomanometer reference measurement, either arm will interfere with the reference device measurement. The Omron wrist cuff measurement was done at least 60 s after the reference measurement but not more than 90 s to avoid increased variability due to expected physiological trends.

### Modulation of blood pressure

Subjects were asked to perform activities that modulated blood pressure to increase the dynamic range of observed SBP and DBP. The lowering of BP was induced by asking the subject to sit with feet raised on a stool or chair and hold a warm water bottle (warm stimulus) in their hand wrapped with an insulating cloth. The increases in BP were induced by asking the subject to walk briskly (mild exercise), as physically able, for about 10 min and holding an ice pack (cold presser) in their hands for 5–10 min as tolerated. Three consecutive recordings of simultaneous and sequential measurements of BP were performed after each of these BP modulating activities.

### Data preparation and processing

The SimpleSense data for each patient was first subjected to a data quality assessment. Segments of data that were of insufficient quality due to the presence of noise due to any movements were removed from further consideration. From the recorded data that was deemed to be of acceptable quality, within a 300-s window preceding a time-stamped observation of SBP and DBP by the observers using the gold standard sphygmomanometer, a 60-s window of data with acceptable quality was extracted and associated with that observation. These steps were performed for each recorded BP value from the gold standard device. Thus, a dataset was prepared with the 60-s-long segments of SimpleSense device data and the associated target SBP and DBP values for training the SimpleSense-BP algorithm. The systolic and diastolic reference measurements acquired from the gold standard device were then randomly split into two sequestered sets of 80%/20% (training/test).

Mean Absolute Difference (MAD) (Eq. 1), Mean Absolute Percentage Difference (MAPD) (Eq. 2), and Root Mean Square Error (RMSE) (Eq. 3) are used to analyze the performance of the models. The statistical aspect of the criteria is discussed in IEEE 1708-2019a.

1 $$MAD=(\sum_{i=1}^{n}|{p}_{i}-{y}_{i}|)/n$$

2 $$MAPD=(\sum_{i=1}^{n}100*|{p}_{i}-{y}_{i}|/{y}_{i})/n$$

3 $$RMSE=\sqrt{\frac{\sum_{i=1}^{n}{({p}_{i}-{y}_{i})}^{2}}{n}}$$

where ${p}_{i}$ is the test device measurement, ${y}_{i}$ is the average of the adjacent two reference measurements taken before and after device measurement as defined in ISO 81060-2:2019, and $n$ is the data size. Applying a constant accuracy limit to measurements at both the low and high ends of BP is biased because the variability may increase at both extremes of BP. An incorrectly significant error may be introduced. Therefore, MAPD is used in addition to MAD.

Further, we evaluated the contribution of the SimpleSense device measured inputs and demographics for BP estimation in comparison to models that use only demographics data. The comparison method follows the recommendation by Mukkamala et al.^16^. The description of the method to compare models described by Natarajan et al.^17^ presents a convenient method to measure overall accuracy through bootstrap RMSE. First, we trained a reference model that uses only the demographics data (Age, Gender, Height, and Weight) on the same training data as SimpleSense-BP, to estimate SBP and DBP. The reference model training procedure used the same training hyperparameters for the automl framework as the SimpleSense-BP model. We took 10,000 random samples of the test set measurements with replacement. The number of samples per iteration was equal to the size of the test set. For each sample, we computed the Root Mean Square Error (RMSE) for each model and the difference between the RMSEs of the two models for comparison as (SimpleSense-BP estimated BP—Demographics only estimated BP for SBP and DBP). The criteria to support the superiority of one model over the other was that the difference in RMSE errors should have an upper 95% Confidence Interval (CI) less than 0 mmHg.

### SimpleSense data analysis and feature exploration

#### ECG features

The ECG features were RR intervals and timing of the R peaks occurrence. The R peaks of the ECG waveform were detected algorithmically. The detected R peaks were used to determine the bounds of the RR intervals for each heartbeat. An ensemble average was computed for one minute of data centered on the measurement of the blood pressure from the reference device.

#### Heart sound features

Heart sound ensembles were defined based on the RR intervals extracted from the ECG waveform. The ensemble average was computed across one minute of data centered at the blood pressure measurement from the reference device like the ECG waveform. The S1 and S2 times of occurrence and root mean square amplitudes were algorithmically extracted from the ensemble average.

#### ECG and heart sound features

**Figure 3:**
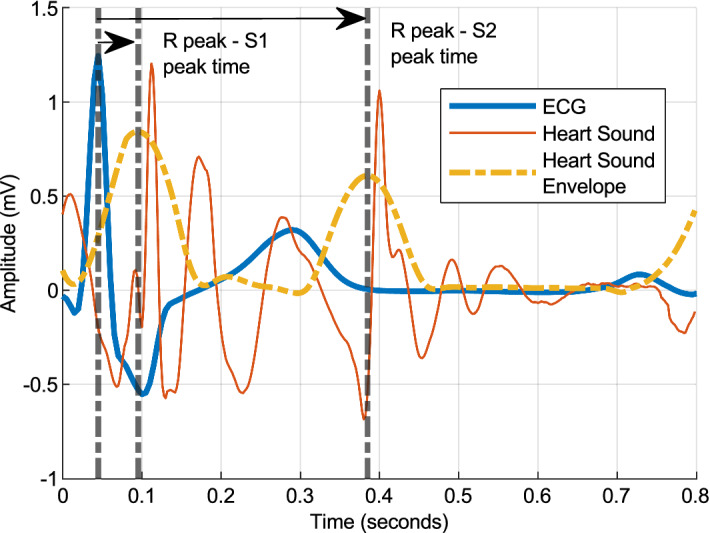
Cross signal features—ECG and Heart sound.

Based on the extracted timing of the R peak of the ECG and the S1 and S2 peaks of the heart sound, a timing feature was extracted. The time elapsed between the R peak and the S1 and S2 sounds is illustrated in Fig. 3. Figure 3 further summarizes the metrics as derived from ECG and heart sound.

Table S1 in the supplementary material provides a list of features, their source, and the descriptions of the features. The SimpleSense device acquires two channels of thoracic impedance, with one channel spanning the thorax from the right shoulder to the lower-left abdomen and the other around the abdomen. Respiration-related features are obtained for each channel and are treated as independent features because of the type of respiration effort measured by the two channels—thoracic and abdominal.

## Results

### Subject characteristics

**Figure 4:**
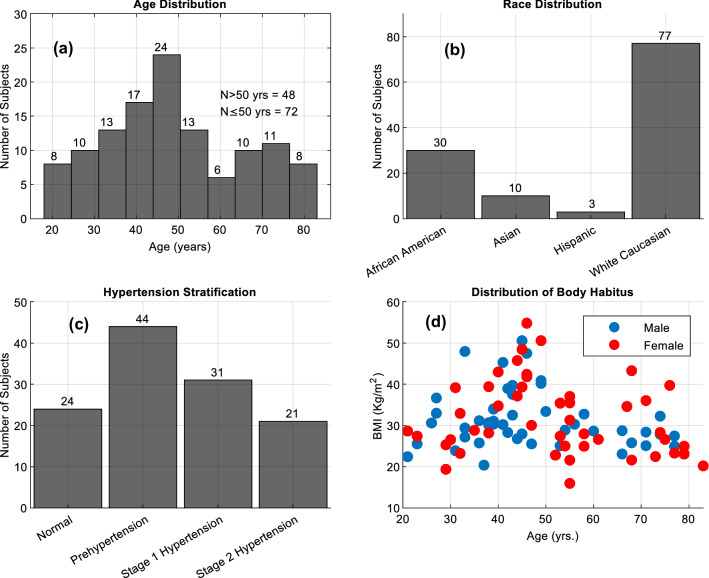
Subject population characteristics.

A total of 120 patients were recruited for the study. The recruited subject population was representative of the adult US census. The age distribution was 48.7 ± 16.8 years, with 48% male. Figure 4 presents (a) the histogram of age, (b) the distribution of race, (c) the hypertension stratifications as per Table 1, and (d) the distribution of age vs. Body Mass Index (BMI). The distribution of age vs. BMI shows the range of body habitus of the participating subjects. Body habitus is a metric that is necessary to analyze for wearable devices such as the SimpleSense device used in this paper.

### Blood pressure data

**Figure 5:**
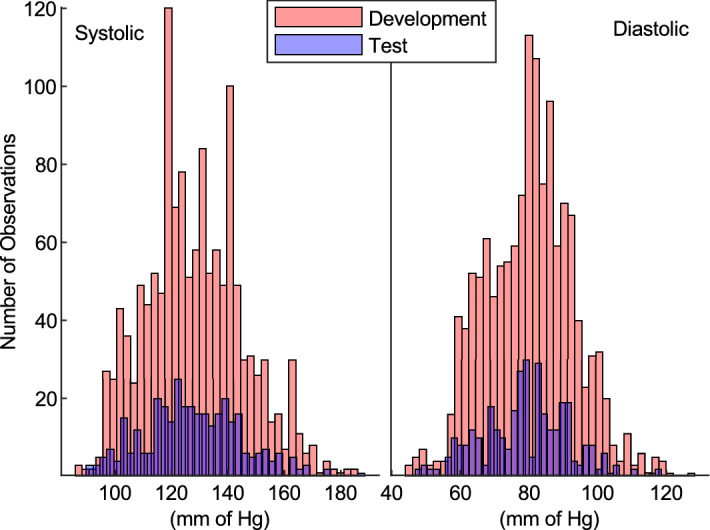
Distribution of sphygmomanometer (gold standard device) measurements.

After the preparation of data collected in the study, the training set had 1348 observations of adequate quality, and the test set had 338 observations of adequate quality for use. Figure 5 shows the distribution of the systolic and diastolic blood pressure values within the data sets.

### Feature selection

**Figure 6:**
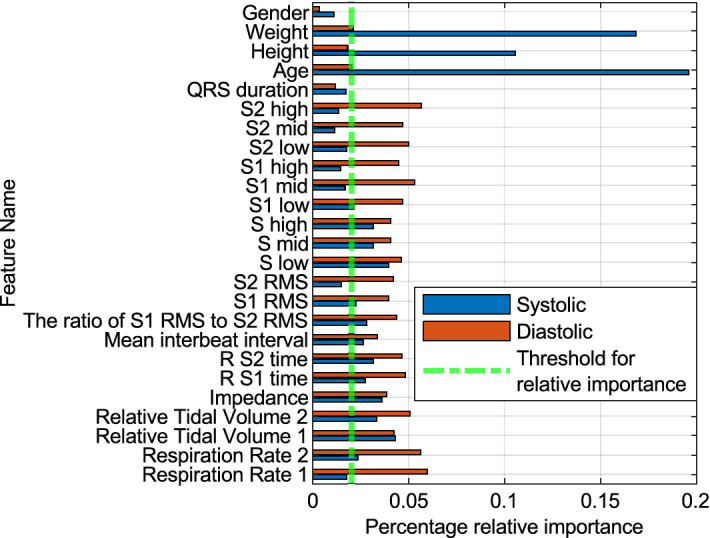
Relative importance of features for predicting SBP and DBP using impurity-based feature importance.

A random forest regression model was trained on the complete list of features in the training dataset for SBP and DBP. Impurity-based feature importance was used to compute feature importance. Features that had more than 2% overall relative importance were selected. Figure 6 shows the list of features and their importance for SBP and DBP. The list of features that were considered important for SBP was – Respiration rate 2, Relative Tidal Volume 1, Relative Tidal Volume 2, impedance, R to S1 time, R to S2 time, Mean interbeat interval, the ratio of S1 RMS to S2 RMS, S low, S mid, S high, QRS duration, age, height, and weight.

Similarly, for DBP—Respiration rate 2, Relative Tidal Volume 1, Relative Tidal Volume 2, impedance, R to S2 time, Mean interbeat interval, the ratio of S1 RMS to S2 RMS, S1 RMS, S low, S mid, S high, S1 low, age, height, and weight. Based on these feature selections, it is evident that demographic data is a strong predictor of SBP and DBP values. However, there is potential benefit in terms of accuracy improvements resulting from using the SimpleSense measured signals in addition to demographics and machine learning methods to find the association with a hemodynamic parameter that can be used to estimate BP. Here, we seek empirical evidence for this potential improvement.

### Performance evaluation

The Systolic and Diastolic models are ensembles of regression trees. The ensemble model was trained using the auto-sklearn framework^18^. With an overall time for a task set to 4 min and individual evaluations set to 30 s, the final diastolic model included eight tree-based regression models, and the systolic model included five regression models.

**Figure 7:**
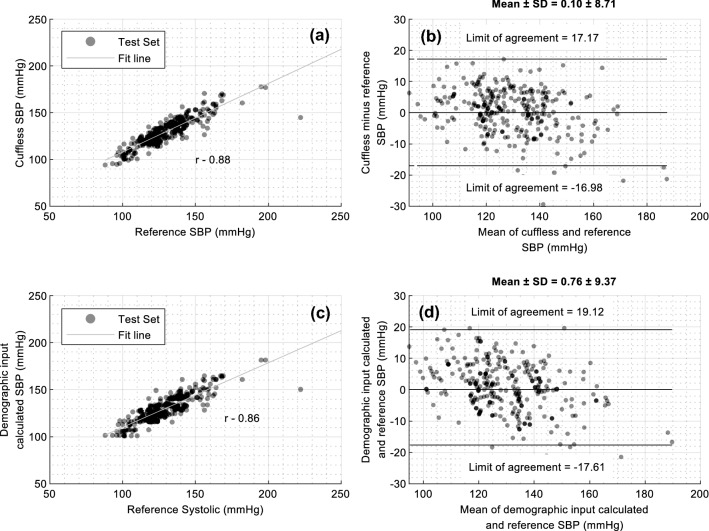
Comparison of actual and predicted measures of Systolic blood pressures from the SimpleSense-BP cuffless model and a baseline model that uses only demographics data. (**a**) scatter plot of predicted vs actual SBP for test set only for SimpleSense-BP device with the correlation coefficient (**b**) Bland Altman plot for test set with limits of agreement and the mean and standard deviation of errors compared to the reference measurement (**c**) scatter plot of predicted vs actual SBP on test set only for baseline model (**d**) Bland Altman plot on test set only with limits of agreement and the mean and standard deviation of errors compared to the reference measurement for the baseline model.

Figure 7 illustrates the performance of the SBP prediction models. The errors in prediction at the higher blood pressure values are greater at higher actual SBPs. The variability of BP at higher SBP is greater and leads to a biased estimate of performance when evaluated separately^14^. Additionally, age-related increases in variability of BP are known^19^.

**Figure 8:**
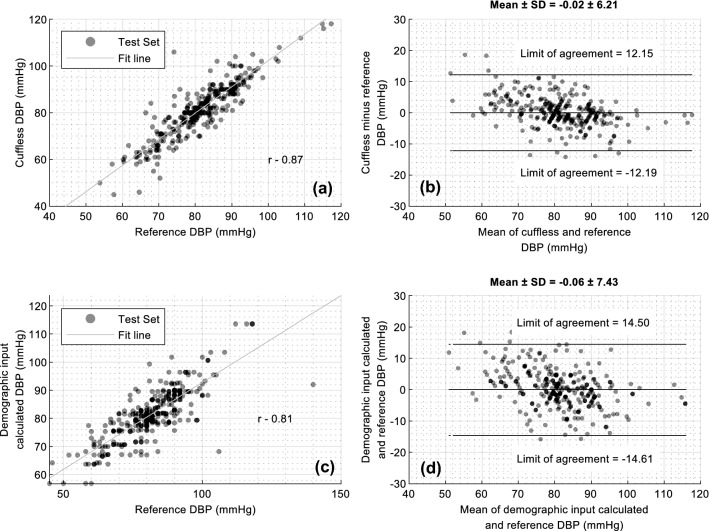
Comparison of actual and predicted measures of Diastolic blood pressures from the SimpleSense-BP cuffless model and a baseline model that uses only demographics data. (**a**) scatter plot of predicted vs actual SBP for test set only for SimpleSense-BP device with the correlation coefficient (**b**) Bland Altman plot for test set with limits of agreement and the mean and standard deviation of errors compared to the reference measurement (**c**) scatter plot of predicted vs actual SBP on test set only for baseline model (**d**) Bland Altman plot on test set only with limits of agreement and the mean and standard deviation of errors compared to the reference measurement for the baseline model.

Figure 8 illustrates the performance of the DBP prediction model. Notably, the dynamic range of the observed DBP values is smaller than SBP. The overestimation of DBP when the actual DBP is below 55 mm of Hg is observable. Although these errors may not lead to a misinterpretation of prehypertensive in the individuals, additional sampling from those with low DBP values would improve the calibrated accuracy of the DBP models.

The results of bootstrap RMSE with the 95% CI are presented in Table 2.

**Table 2:** Results of the bootstrap RMSE analysis for each model SimpleSense-BP, baseline model using demographics data only, and the difference between the paired measurements of RMSE between the models.

Measurement type	SimpleSense-BP (RMSE—mmHg)	Baseline model (RMSE—mmHg)	SimpleSense-BP—baseline model (RMSE—mmHg)
SBP	8.62 (6.87, 11.12)	9.33 (7.82, 11.74)	− 0.72 (− 1.32, − 0.14) (p < 0.0057)
DBP	6.15 (4.87, 7.71)	7.38 (6.19, 8.78)	− 1.23 (− 1.74, − 0.74) (p < 0.0001)

The upper 95% CI for the difference between the SimpleSense-BP and the baseline model for both SBP and DBP are below zero with the differences in RMSE calculated as (SimpleSense-BP—baseline model). With this result, we conclude that the SimpleSense-BP model has a statistically significant improvement in performance over a model that uses demographic data only. We calculated the p-value as the percentile value at which ≥ 0 mmHg difference in RMSE was observed (SBP p < 0.0057; DBP p < 0.0001). Table 3 summarizes the results of the training and testing done on a test set that was sequestered from the training set.

**Table 3:** Performance evaluation of Blood pressures measured by SimpleSense and a wrist cuff device.

Measurement condition	SimpleSense-BP				Wrist Cuff	
	MAD (mm of Hg)		MADP (%)		MAD (mm of Hg)	MADP (%)
	Training	Test	Training	Test		
**Overall**						
Systolic	5.86	6.40	4.56	5.05	8.93	6.69
Diastolic	2.51	5.10	3.26	6.79	6.87	8.39
**Static**						
Systolic	6.72	5.78	4.93	4.55	9.71	6.98
Diastolic	3.82	4.23	4.91	5.53	7.31	8.37
**Warm stimulus and rest (BP lowering)**						
Systolic	5.27	6.42	4.06	4.91	9.62	7.18
Diastolic	3.97	4.23	5.00	5.54	8.31	9.75
**Cold stimulus and walking (BP increasing)**						
Systolic	6.02	5.94	4.58	4.59	10.72	7.62
Diastolic	4.02	2.97	5.12	3.67	7.46	8.48
**Rest**						
Systolic	6.16	5.90	4.73	4.46	8.83	6.62
Diastolic	4.04	4.01	5.12	5.28	7.5	8.69

The test protocol includes induced variation of blood pressure within an individual participant using a cold pressor test, heating of extremities, relaxed posture, and brisk exercise in the form of walking. Further, bootstrap resampling was done for the systolic BP model and Diastolic BP model to obtain a 95% confidence interval for MAD values. We performed 1000 iterations of resampling with replacement with each sample size equal to the test set size and the confidence intervals for the systolic and diastolic MAD were found to be (5.36, 6.85 mm Hg) and (3.51, 4.65 mmHg), respectively.

## Discussion

Two ensemble models were trained on the data collected from 120 subjects. The two models predict systolic and diastolic pressures based on features extracted from the SimpleSense data and demographics data, inclusive of Age, Gender, Height, and Weight. This approach would be consistent with the class of BP measurement called cuffless BP monitors. There are two novel aspects reported herein—*First*, the use of heart sounds, ECG, and thoracic impedance as inputs to estimate SBP and DBP, and *second*, the size of the subject population for the evaluation of such a cuffless approach is the largest to date in the literature. The device and model are cuffless and do not require a cuff calibration procedure. This removes the burden on the user to calibrate the device and eliminates errors due to the wrong calibration. The performance observed in this study supports a determination that this approach is indeed feasible for monitoring BP of adults without a diagnosed arrhythmia or actively taking vasoactive anti-hypertensive medications. It is further observed that performance may improve at the low and high BP ranges of measurement by increasing the number of subjects in those stratifications. However, as a generalized approach that should be reliable for the US population, the presented model meets the criteria for required performance as per the current IEEE 1708 standard. Recent work by Mukkamala et al. has revealed limitations to the approaches described herein. The measurement of errors in BP must be done in a longitudinal study with tracking of BP over time for the same individual. This should be a part of the evaluation of new BP estimation methods. There are practical challenges in the recruitment of patients to meet the criteria for the subject population outlined in the latest amendment to IEEE 1708a 2019; the standard requires a separate study for age groups over 50 yrs., but requires recruitment of at least 21 subjects overall in each stratification of BP. Finding subjects under 50 years of age with stage 2 hypertension is challenging regardless of the resources available because we face the ethical conflict of not immediately recommending their treatment with vasoactive drugs, and subjects under vasoactive drug treatment must be excluded. This study explored the possibility of including a broader population in terms of age, and we present the results with age-stratified accuracy presented in Tables S4 and S5 of the supplementary materials. Notably, we observed that the age group of ≥ 68 years had an error rate for SBP of 8.42 mmHg MAD. The subjects in this age range were 19 (15.83% of the population). Therefore, a larger number of subjects in this age range is warranted to improve the performance of SimpleSense-BP.

The BP estimation method presented herein was not developed to monitor BP in ambulatory patients while performing activities involving movements. SimpleSense is wearable, and estimates of BP can be calculated during movements. However, since the gold standard sphygmomanometer and existing ABPM devices do not claim BP during movements, the training and testing data were collected with the subjects in a stationary state. A claim justifying truly ambulatory BP monitoring using SimpleSense would need further clinical study. Similarly, the current research that is presented emphasizes the algorithm that is developed to predict blood pressure. The algorithm was trained and validated based on the data presented in this article. It should be noted that an independent evaluation and validation would be required before the device can be fully incorporated into commercial applications.

## Supplementary Information

Supplementary Information.

## Data Availability

The datasets generated during and/or analysed during the current study are not publicly available due to the presence of Personally Identifiable Information (PII) for the participating subjects. The data are available from the corresponding author on reasonable request with the understanding that additional processing is required to adequately deidentify the information. All authors reviewed and approved the manuscript.
