# Supplementary material for: Multiparametric cloth-based wearable, SimpleSense, estimates blood pressure
Source: Sci Rep. 2022 Jul 29;12:13059. doi: 10.1038/s41598-022-17223-x (PMC9338290; doi:10.1038/s41598-022-17223-x)
Supplement: Supplementary file 1 — Supplementary Information. [file 41598_2022_17223_MOESM1_ESM.docx]

# Supplementary Material

## Description of features

**Table S 1 List of features, descriptions, and evidence correlating feature and SBP and DBP in the literature**

| **#** | **Name** | **Source** | **Description** | **Studies in the literature that have correlated features to either SBP or DBP, or both** |
| --- | --- | --- | --- | --- |
| 1 | Mean respiration rate 1 | Impedance | Mean respiration rate computed over a minute of thoracic impedance data from channel 1 | Respiration is known to influence BP^1-3^. Slow breathing may result in potential improvement for patients with essential hypertension^4^. |
| 2 | Mean respiration rate 2 |  | Mean respiration rate computed over a minute of thoracic impedance data from channel 2 |  |
| 3 | Relative Tidal Volume 1 |  | Mean of the range of thoracic impedance values measured within a minute of SimpleSense data from channel 1. These ranges are computed over 30-second segments with 50 % overlap. |  |
| 4 | Relative Tidal Volume 2 |  | Mean of the range of thoracic impedance values measured within a minute of SimpleSense data from channel 2. These ranges are computed over 30-second segments with 50 % overlap. |  |
| 5 | Mean thoracic impedance 1 |  | Mean of the thoracic impedance values from channel one over one minute. | Thoracic impedance is a non-invasive method of assessing fluid status and hemodynamics. The potential utility of impedance and its first derivative impedance cardiography in the hemodynamic assessments of patients with heart failure is prevalent in the literature^5^. |
| 6 | R to S1 time | Heart sound and ECG | Mean of the time intervals between the occurrence of the R peak in the ECG and the S1 heart sound peak in the Heart sound signal over a minute of data. | These timing parameters are scarcely explored in literature due to the scarcity of simultaneous measurement of heart sounds and ECG. From a physiological standpoint, R-S1 and R-S2 timings are reflective of the systolic and diastolic timings encompassing the pre-ejection time. |
| 7 | R to S2 time |  | Mean of the time intervals between the occurrence of the R peak in the ECG and the S2 heart sound peak in the Heart sound signal over a minute of data. |  |
| 8 | Mean interbeat interval | Heart sound | Average interval in seconds between heartbeats. Both ECG and Heart sounds are used to compute the mean heart rate. | Heart rate and heart rate variability are known modulators of blood pressure, and their non-linear coupling relationship is yet to be established^6,7^. |
| 9 | The ratio of S1 RMS to S2 RMS |  | The ratio of S1 RMS and S2 RMS | A novel feature explored in this study. |
| 10 | S1 RMS |  | Mean of the root mean square (RMS) amplitude over a 125-millisecond window centered at the time of occurrence of the S1 sound of the heart sound. A single value, the mean of the RMS, is computed from a minute of heart sound signal. | There are several studies in the literature that have sought to associate characteristic heart sound amplitudes with SBP and DBP. The list of features explored in this paper is an aggregation of all the reported features in the literature^8,9^. |
| 11 | S2 RMS |  | Mean of the root mean square (RMS) amplitude over a 125-millisecond window centered at the time of occurrence of the S2 sound of the heart sound. A single value, the mean of the RMS, is computed from a minute of heart sound signal. |  |
| 12 | S low |  | Sum of amplitudes in the frequencies from 50 to 100 Hz relative to total amplitude across all frequencies |  |
| 13 | S mid |  | Sum of amplitudes in the frequencies from 125 to 175 Hz relative to total amplitude across all frequencies |  |
| 14 | S high |  | Sum of amplitudes in the frequencies from 175 to 250 Hz relative to total amplitude across all frequencies |  |
| 15 | S1 low |  | Sum of amplitudes in the frequencies from 50 to 100 Hz relative to total amplitude across all frequencies for a sequence of 125 milliseconds centered on the S1 peak sound |  |
| 16 | S1 mid |  | Sum of amplitudes in the frequencies from 125 to 175 Hz relative to total amplitude across all frequencies for a sequence of 125 milliseconds centered on the S1 peak sound |  |
| 17 | S1 high |  | Sum of amplitudes in the frequencies from 175 to 250 Hz relative to total amplitude across all frequencies for a sequence of 125 milliseconds centered on the S1 peak sound |  |
| 18 | S2 low |  | Sum of amplitudes in the frequencies from 50 to 100 Hz relative to total amplitude across all frequencies for a sequence of 125 milliseconds centered on the S2 peak sound |  |
| 19 | S2 mid |  | Sum of amplitudes in the frequencies from 125 to 175 Hz relative to total amplitude across all frequencies for a sequence of 125 milliseconds centered on the S2 peak sound |  |
| 20 | S2 high |  | Sum of amplitudes in the frequencies from 175 to 250 Hz relative to total amplitude across all frequencies for a sequence of 125 milliseconds centered on the S2 peak sound |  |
| 21 | QRS duration | ECG | Number of samples of the ECG data between the first zero crossing before QRS complex and first zero crossing after the QRS complex - denotes the demarcation of the QRS complex | Mixed evidence is reported on the association between QRS duration and blood pressure^10^. In this paper, empirical evaluation of the utility of QRS duration was explored. QRS duration was found to be unimportant as a predictor for both SBP and DBP. |
| 22 | Age | Demographics | Age of the subject | The correlation between age, gender, BMI, and BP has been established in several studies^11-13^. |
| 23 | Height | Demographics | Height of the subject |  |
| 24 | Weight | Demographics | Weight of the subject |  |
| 25 | Gender | Demographics | Gender of the subject |  |

## Procedure For Study Data Collection

Note to technician/Observers: - Subject is requested to wear a front button shirt. The SimpleSense garment is to be worn on bare skin. The subject wears a shirt on top of the SimpleSense garment.

The subject should not have had a meal 30 minutes prior to coming in for testing.

The technician will be wearing a face mask, safety glasses, hairnet, face shield, hand gloves, lab coat. The gloves will be replaced after every test.

1. The subject comes in and sits in the lobby, and signs the consent form (10-15 minutes)
2. The subject is asked for their age, height, and weight (approx.), medication history, how they are feeling (relaxed or stressed) when the subject had the last meal.
3. Subject moves to the conference room (sequestered room for privacy), and the following steps are performed:
   1. Subject removes shirt and inner vest/bra
   2. The subject is asked to wear the SimpleSense SAU and garment as per instructions. The subject can opt for help from the technician
   3. The subject is made to sit on an armchair with their left arm resting on the armrest of the chair.
   4. A blood pressure arm cuff is attached to the left arm of the subject as per instructions. The site of cuff attachment should be at the level of the left ventricle. The cuff is applied to the bare arm, and there should be no arm compression in the proximity of the cuff.
   5. A wrist blood pressure cuff is attached to the left wrist as per instructions. The cuff is applied to the bare wrist, and there should be no arm compression in the proximity of the cuff.
   6. A handheld IR temperature monitor is used to take body temperature from the forehead.
   7. A pulse oximeter is attached to the right index finger and switched on
4. During the testing, the subject is asked to sit still and be silent. **The subject should avoid speaking during the procedure.**
5. The SimpleSense recording is initiated, and signal quality tested
6. The SimpleSense data recording is initiated, and start time is noted from the mobile phone clock
7. The subjects are asked to relax for 10 minutes with their arm hanging down loosely. The subject should sit comfortably with feet flat on the floor. The subject should have back elbow, elbow, and forearm support.
8. Record the SpO_2_ value from the oximeter. Go to the symptoms log, select "**other,**" and enter the data in the format "s <enter percentage value>."
9. Record temperature. Go to the symptoms log, select "**other,**" and enter the data in the format "t <enter temperature in degrees Fahrenheit>."
10. Three measurements are taken from the arm cuff and the wrist cuff to obtain static blood pressure. The blood pressure reading will be taken in the following sequence:

- The blood pressure is measured from the blood pressure arm cuff. The observers shall use the Korotkoff sound [fifth phase (K5)] for determining the REFERENCE DIASTOLIC BLOOD PRESSURE. If the Korotkoff sound [fifth phase (K5)] for determining REFERENCE DIASTOLIC BLOOD PRESSURE is not audible, the subject shall be excluded. Note systolic and diastolic blood pressure and heart rate. Go to the symptoms log, select "**other**" and enter the data in the format "sa <systolic value/diastolic value> hr <heart rate value>".

- The blood pressure is measured from the blood pressure wrist cuff. Note systolic and diastolic blood pressure and heart rate. Go to the symptoms log, select "**other**" and enter the data in the format "sw <systolic value/diastolic value> hr <heart rate value>".

There should be at least 60 seconds difference between 2 consecutive readings.

Two observers shall simultaneously determine the SYSTOLIC BLOOD PRESSURE and DIASTOLIC BLOOD PRESSURE on each subject using a double stethoscope. Any pair of observers' SYSTOLIC BLOOD PRESSURE VALUE or DIASTOLIC BLOOD PRESSURE VALUE with a difference greater than four mmHg (0,53 kPa) shall be excluded.

Unless the SPHYGMOMANOMETER-UNDER-TEST is intended for use during significantly irregular heart rhythm, if either observer detects significantly irregular heart rhythm, that reading shall be excluded.

$$p_{REF i}=\frac{p_{REF i,1}+p_{REF i,2}}{2}$$

where

*p*_REFi ,1_ is the BLOOD PRESSURE determined by observer 1 for the i^th^ reading;

*p*_REFi ,2_ is the BLOOD PRESSURE determined by observer 2 for the i^th^ reading;

*p*_REFi_ is the REFERENCE BLOOD PRESSURE value for the i^th^ reading.

If any readings are excluded, additional pair(s) of readings shall be taken to ensure that the required number of valid test-REFERENCE pairs are available.

1. The subjects are asked to relax for 10 minutes with their feet up, resting on a tool or a chair. This is an attempt at reducing blood pressure. The subject are asked to hold a hand warmer, and the hands are wrapped with an insulating cloth for 10 minutes.
2. Record the SpO_2_ value from the oximeter and note the time from the mobile phone clock.
3. Three pairs of measurements are taken from the arm cuff and the wrist cuff. The blood pressure reading will be taken in the following sequence:

- The blood pressure is measured from the blood pressure arm cuff. The observers shall use the Korotkoff sound [fifth phase (K5)] for determining the REFERENCE DIASTOLIC BLOOD PRESSURE. If the Korotkoff sound [fifth phase (K5)] for determining REFERENCE DIASTOLIC BLOOD PRESSURE is not audible, the subject shall be excluded. Note systolic and diastolic blood pressure and heart rate. Go to the symptoms log, select "**other**" and enter the data in the format "va <systolic value/diastolic value> hr <heart rate value>".

- The blood pressure is measured from the blood pressure wrist cuff. Note systolic and diastolic blood pressure and heart rate. Go to the symptoms log, select "**other**" and enter the data in the format "vw <systolic value/diastolic value> hr <heart rate value>".

There should be at least 60 seconds difference between 2 consecutive readings. For each pair of readings, observe the blood pressure variation from the **static blood pressure values** and record them.

Two observers shall simultaneously determine the SYSTOLIC BLOOD PRESSURE and DIASTOLIC BLOOD PRESSURE on each subject using a double stethoscope. Any pair of observers' SYSTOLIC BLOOD PRESSURE VALUE or DIASTOLIC BLOOD PRESSURE VALUE with a difference greater than four mmHg (0,53 kPa) shall be excluded.

Unless the SPHYGMOMANOMETER-UNDER-TEST is intended for use during significantly irregular heart rhythm, if either observer detects significantly irregular heart rhythm, that reading shall be excluded.

$$p_{REF i}=\frac{p_{REF i,1}+p_{REF i,2}}{2}$$

where

*p*_REFi ,1_ is the BLOOD PRESSURE determined by observer 1 for the i^th^ reading;

*p*_REFi ,2_ is the BLOOD PRESSURE determined by observer 2 for the i^th^ reading;

*p*_REFi_ is the REFERENCE BLOOD PRESSURE value for the i^th^ reading.

If any readings are excluded, additional pair(s) of readings shall be taken to ensure that the required number of valid test-REFERENCE pairs are available.

1. The subjects are asked to do a brisk walk for 5-10 minutes (based on their fitness level). The subjects are asked to hold in their hands an ice pack for 5 - 10 minutes. This is an attempt at increasing blood pressure.
2. Record the SpO_2_ value from the oximeter and note the time from the mobile phone clock.
3. Three pairs of measurements are taken from the arm cuff and the wrist cuff. The blood pressure reading will be taken in the following sequence:

- The blood pressure is measured from the blood pressure arm cuff. The Korotkoff sound [fifth phase (K5)] shall be used by the observers for determining the REFERENCE DIASTOLIC BLOOD PRESSURE. If the Korotkoff sound [fifth phase (K5)] for determining REFERENCE DIASTOLIC BLOOD PRESSURE is not audible, the subject shall be excluded. Note systolic and diastolic blood pressure and heart rate. Go to the symptoms log, select "**other**" and enter the data in the format "va <systolic value/diastolic value> hr <heart rate value>".

- The blood pressure is measured from the blood pressure wrist cuff. Note systolic and diastolic blood pressure and heart rate. Go to the symptoms log, select "**other**" and enter the data in the format "vw <systolic value/diastolic value> hr <heart rate value>".

There should be at least 60 seconds difference between 2 consecutive readings. For each pair of readings, observe the blood pressure variation from the **static blood pressure values** and record them.

Two observers shall simultaneously determine the SYSTOLIC BLOOD PRESSURE and DIASTOLIC BLOOD PRESSURE on each subject using a double stethoscope. Any pair of observers' SYSTOLIC BLOOD PRESSURE VALUE or DIASTOLIC BLOOD PRESSURE VALUE with a difference greater than four mmHg (0,53 kPa) shall be excluded.

Unless the SPHYGMOMANOMETER-UNDER-TEST is intended for use during significantly irregular heart rhythm, if either observer detects significantly irregular heart rhythm, that reading shall be excluded.

$$p_{REF i}=\frac{p_{REF i,1}+p_{REF i,2}}{2}$$

where

*p*_REFi ,1_ is the BLOOD PRESSURE determined by observer 1 for the i^th^ reading;

*p*_REFi ,2_ is the BLOOD PRESSURE determined by observer 2 for the i^th^ reading;

*p*_REFi_ is the REFERENCE BLOOD PRESSURE value for the i^th^ reading.

If any readings are excluded, additional pair(s) of readings shall be taken to ensure that the required number of valid test-REFERENCE pairs are available.

**Table - Requirements on the induced blood pressure changes ^a^**

|  | **Changes of blood pressure from the point of calibration (mmHg)** | | | |
| --- | --- | --- | --- | --- |
| **Systolic blood pressure** | -30 – -15 | -15 – 0 | 0 – 15 | 15 – 30 |
| **Diastolic blood pressure** | -20 – -10 | -10 – 0 | 0 – 10 | 10 – 20 |
| **Required percentage of samples(at least)** | 13.6% | 34.1% | 34.1% | 13.6% |
| **^a^** Blood pressure change refers to the reference reading measured by the observers minus the value of at the calibration point | | | | |

1. The subjects are asked to relax in an armchair for 15 minutes with their arm hanging down loosely and feet flat on the ground.
2. Record the SpO_2_ value from the oximeter and note the time from the mobile phone clock
3. Three pairs of measurements are taken from the arm cuff and the wrist cuff. The blood pressure reading will be taken in the following sequence:

- The blood pressure is measured from the blood pressure arm cuff. The Korotkoff sound [fifth phase (K5)] shall be used by the observers for determining the REFERENCE DIASTOLIC BLOOD PRESSURE. If the Korotkoff sound [fifth phase (K5)] for determining REFERENCE DIASTOLIC BLOOD PRESSURE is not audible, the subject shall be excluded. Note systolic and diastolic blood pressure and heart rate. Go to the symptoms log, select "**other**" and enter the data in the format "ra <systolic value/diastolic value> hr <heart rate value>".

- The blood pressure is measured from the blood pressure wrist cuff. Note systolic and diastolic blood pressure and heart rate. Go to the symptoms log, select "**other**" and enter the data in the format "rw <systolic value/diastolic value> hr <heart rate value>".

There should be at least 60 seconds difference between 2 consecutive readings.

Two observers shall simultaneously determine the SYSTOLIC BLOOD PRESSURE and DIASTOLIC BLOOD PRESSURE on each subject using a double stethoscope. Any pair of observers' SYSTOLIC BLOOD PRESSURE VALUE or DIASTOLIC BLOOD PRESSURE VALUE with a difference greater than four mmHg (0,53 kPa) shall be excluded.

Unless the SPHYGMOMANOMETER-UNDER-TEST is intended for use during significantly irregular heart rhythm, if either observer detects significantly irregular heart rhythm, that reading shall be excluded.

$$p_{REF i}=\frac{p_{REF i,1}+p_{REF i,2}}{2}$$

where

*p*_REFi ,1_ is the BLOOD PRESSURE determined by observer 1 for the i^th^ reading;

*p*_REFi ,2_ is the BLOOD PRESSURE determined by observer 2 for the i^th^ reading;

*p*_REFi_ is the REFERENCE BLOOD PRESSURE value for the i^th^ reading.

If any readings are excluded, additional pair(s) of readings shall be taken to ensure that the required number of valid test-REFERENCE pairs are available.

## Blood Pressure model descriptions

### Systolic Model

**Table S 2 Systolic Blood Pressure Estimation Ensemble of regression models**

| **Rank** | **Weight** | **Model Type** | **Model Parameters – (sklearn regressor)** |
| --- | --- | --- | --- |
| 1 | 0.78 | Gradient Boosting | - Loss function to be optimized = least squares - Learning rate shrinks the contribution of each tree by learning_rate = 0.22526525740632317 - The number of boosting stages to perform = 100 - The fraction of samples to be used for fitting the individual base learners = 1.0 - The function to measure the quality of a split = friedman_mse - The minimum number of samples required to split an internal node=8 - The minimum number of samples required to be at a leaf node=17 - The minimum weighted fraction of the sum total of weights (of all the input samples) required to be at a leaf node=0.0. - Maximum depth of the individual regression estimators. The maximum depth limits the number of nodes in the tree=None. - A node will be split if this split induces a decrease of the impurity greater than or equal to this value=0.0. - An estimator object that is used to compute the initial predictions=None. - The fraction of total number of features to consider when looking for the best split=0.7683292269455733 - Grow trees with max_leaf_nodes in best-first fashion=None. - Do not reuse the solution of the previous call to fit and add more estimators to the ensemble - The proportion of training data to set aside as validation set for early stopping=0.35101002117752517. - n_iter_no_change is used to decide if early stopping will be used to terminate training when validation score is not improving=None - Tolerance for the early stopping=1e-07. - Complexity parameter used for Minimal Cost-Complexity Pruning=0.0. |
| 2 | 0.1 | K nearest neighbors | - Number of neighbors to use by default=1 - Weight function used in prediction = ‘uniform’. - Algorithm used to compute the nearest neighbors=’auto’ - Power parameter for the Minkowski metric=1. - The distance metric to use for the tree=’minkowski’ |
| 3 | 0.06 | Support vector machine regressor | - Specifies the kernel type to be used in the algorithm='rbf' - Degree of the polynomial kernel function (‘poly’)=3 - Kernel coefficient for ‘rbf’, ‘poly’ and ‘sigmoid’=0.20113065159176252 - Tolerance for stopping criterion=0.0360184306323898. - Regularization parameter=194.03096694114694. - Epsilon in the epsilon-SVR model. It specifies the epsilon-tube within which no penalty is associated in the training loss function with points predicted within a distance epsilon from the actual value=0.0010647224198079256. - Whether to use the shrinking heuristic=True, use shrinkage. - Specify the size of the kernel cache=200MB - Hard limit on iterations within solver=-1 |
| 4 | 0.04 | Adaboost regressor | - The base estimator from which the boosted ensemble is built=None. - The maximum number of estimators at which boosting is terminated=217. - Weight applied to each regressor at each boosting iteration=0.8974499672625733. - The loss function to use when updating the weights after each boosting iteration=’exponential’. |
| 5 | 0.02 | Random forest regressor | - The number of trees in the forest=100. - The function to measure the quality of a split=friedman_mse. - The maximum depth of the tree=None. - The minimum number of samples required to split an internal node=20 - The minimum number of samples required to be at a leaf node=11 - The minimum weighted fraction of the sum total of weights (of all the input samples) required to be at a leaf node=0.0. - The fraction of the number of features to consider when looking for the best split=0.7640742005089622 - Grow trees with max_leaf_nodes in best-first fashion=None. - A node will be split if this split induces a decrease of the impurity greater than or equal to this value=0.0. - Whether bootstrap samples are used when building trees=True. - Do not reuse the solution of the previous call to fit and add more estimators to the ensemble. - Complexity parameter used for Minimal Cost-Complexity Pruning=0.0 - the number of samples to draw from X to train each base estimator= All samples |

### Diastolic Model

**Table S 3 Diastolic Blood Pressure Estimation Ensemble of regression models**

| **Rank** | **Weight** | **Model Type** | **Model Parameters – (sklearn regressor)** |
| --- | --- | --- | --- |
| 1 | 0.22 | Gradient Boosting | - Loss function to be optimized=least squares. - Learning rate shrinks the contribution of each tree by learning_rate= 0.11877384799096732 - The number of boosting stages to perform=100 - The fraction of samples to be used for fitting the individual base learners=1.0. - The function to measure the quality of a split= friedman_mse. - The minimum number of samples required to split an internal node=2 - The minimum number of samples required to be at a leaf node=10. - The minimum weighted fraction of the sum total of weights (of all the input samples) required to be at a leaf node=0.0. - Maximum depth of the individual regression estimators. The maximum depth limits the number of nodes in the tree=None. - A node will be split if this split induces a decrease of the impurity greater than or equal to this value=0.0. - An estimator object that is used to compute the initial predictions=None. - The number of features to consider when looking for the best split=None - Grow trees with max_leaf_nodes in best-first fashion=43. - Donot reuse the solution of the previous call to fit and add more estimators to the ensemble. - The proportion of training data to set aside as validation set for early stopping= 0.2841571323040151. - n_iter_no_change is used to decide if early stopping will be used to terminate training when validation score is not improving=12. - Tolerance for the early stopping=1e-07. - Complexity parameter used for Minimal Cost-Complexity Pruning=0.0. |
| 2 | 0.2 | Gradient Boosting | - Loss function to be optimized=least squares. - Learning rate shrinks the contribution of each tree by learning_rate= 0.04857791763352136 - The number of boosting stages to perform=100 - The fraction of samples to be used for fitting the individual base learners=1.0. - The function to measure the quality of a split=friedman_mse. - The minimum number of samples required to split an internal node=2 - The minimum number of samples required to be at a leaf node=7. - The minimum weighted fraction of the sum total of weights (of all the input samples) required to be at a leaf node=0.0. - Maximum depth of the individual regression estimators. The maximum depth limits the number of nodes in the tree=None. - A node will be split if this split induces a decrease of the impurity greater than or equal to this value=0.0. - An estimator object that is used to compute the initial predictions=None. - The number of features to consider when looking for the best split=None. - Grow trees with max_leaf_nodes in best-first fashion=1799. - Do not reuse the solution of the previous call to fit and add more estimators to the ensemble. - The proportion of training data to set aside as validation set for early stopping=0.1. - n_iter_no_change is used to decide if early stopping will be used to terminate training when validation score is not improving=7. - Tolerance for the early stopping=1e-07. - Complexity parameter used for Minimal Cost-Complexity Pruning=0.0. |
| 3 | 0.14 | Gradient Boosting | - Loss function to be optimized=least squares. - Learning rate shrinks the contribution of each tree by learning_rate= 0.046029669658888245 - The number of boosting stages to perform=100 - The fraction of samples to be used for fitting the individual base learners=1.0. - The function to measure the quality of a split=friedman_mse. - The minimum number of samples required to split an internal node=2 - The minimum number of samples required to be at a leaf node=7. - The minimum weighted fraction of the sum total of weights (of all the input samples) required to be at a leaf node=0.0. - Maximum depth of the individual regression estimators. The maximum depth limits the number of nodes in the tree=None. - A node will be split if this split induces a decrease of the impurity greater than or equal to this value=0.0. - An estimator object that is used to compute the initial predictions=None. - The number of features to consider when looking for the best split=None. - Grow trees with max_leaf_nodes in best-first fashion=27. - Do not reuse the solution of the previous call to fit and add more estimators to the ensemble. - The proportion of training data to set aside as validation set for early stopping=0.1. - n_iter_no_change is used to decide if early stopping will be used to terminate training when validation score is not improving=18. - Tolerance for the early stopping=1e-07. - Complexity parameter used for Minimal Cost-Complexity Pruning=0.0. |
| 4 | 0.14 | Gradient Boosting | - Loss function to be optimized=least squares. - Learning rate shrinks the contribution of each tree by learning_rate= 0.16896088251156324 - The number of boosting stages to perform=100 - The fraction of samples to be used for fitting the individual base learners=1.0. - The function to measure the quality of a split=friedman_mse. - The minimum number of samples required to split an internal node=2 - The minimum number of samples required to be at a leaf node=14. - The minimum weighted fraction of the sum total of weights (of all the input samples) required to be at a leaf node=0.0. - Maximum depth of the individual regression estimators. The maximum depth limits the number of nodes in the tree=None. - A node will be split if this split induces a decrease of the impurity greater than or equal to this value=0.0. - An estimator object that is used to compute the initial predictions=None. - The number of features to consider when looking for the best split=None - Grow trees with max_leaf_nodes in best-first fashion=8. - Do not reuse the solution of the previous call to fit and add more estimators to the ensemble. - The proportion of training data to set aside as validation set for early stopping=0.1. - n_iter_no_change is used to decide if early stopping will be used to terminate training when validation score is not improving=None. - Tolerance for the early stopping=1e-07. - Complexity parameter used for Minimal Cost-Complexity Pruning=0.0. |
| 5 | 0.14 | K nearest neighbors | - Number of neighbors to use by default=5 - Weight function used in prediction=’uniform’ - Algorithm used to compute the nearest neighbors=’auto’ - Power parameter for the Minkowski metric=2. - The distance metric to use for the tree=’minkowski’. |
| 6 | 0.08 | Gradient Boosting | - Loss function to be optimized=least squares. - Learning rate shrinks the contribution of each tree by learning_rate= 0.022613964943578323 - The number of boosting stages to perform=100 - The fraction of samples to be used for fitting the individual base learners=1.0 - The function to measure the quality of a split=friedman_mse. - The minimum number of samples required to split an internal node=2 - The minimum number of samples required to be at a leaf node=7. - The minimum weighted fraction of the sum total of weights (of all the input samples) required to be at a leaf node=0.0. - Maximum depth of the individual regression estimators. The maximum depth limits the number of nodes in the tree=None. - A node will be split if this split induces a decrease of the impurity greater than or equal to this value=0.0. - An estimator object that is used to compute the initial predictions=None. - The number of features to consider when looking for the best split=None - Grow trees with max_leaf_nodes in best-first fashion=24. - Do not reuse the solution of the previous call to fit and add more estimators to the ensemble. - The proportion of training data to set aside as validation set for early stopping=0.1. - n_iter_no_change is used to decide if early stopping will be used to terminate training when validation score is not improving=18. - Tolerance for the early stopping=1e-07. - Complexity parameter used for Minimal Cost-Complexity Pruning=0.0. |
| 7 | 0.06 | Gradient Boosting | - Loss function to be optimized=least squares. - Learning rate shrinks the contribution of each tree by learning_rate= 0.03079762265599878 - The number of boosting stages to perform=100 - The fraction of samples to be used for fitting the individual base learners=1.0. - The function to measure the quality of a split=friedman_mse. - The minimum number of samples required to split an internal node=2 - The minimum number of samples required to be at a leaf node=36. - The minimum weighted fraction of the sum total of weights (of all the input samples) required to be at a leaf node=0.0. - Maximum depth of the individual regression estimators. The maximum depth limits the number of nodes in the tree=None. - A node will be split if this split induces a decrease of the impurity greater than or equal to this value=0.0. - An estimator object that is used to compute the initial predictions=None. - The number of features to consider when looking for the best split=None - Grow trees with max_leaf_nodes in best-first fashion=331. - Do not reuse the solution of the previous call to fit and add more estimators to the ensemble. - The proportion of training data to set aside as validation set for early stopping= 0.23608035974923702. - n_iter_no_change is used to decide if early stopping will be used to terminate training when validation score is not improving=9. - Tolerance for the early stopping=1e-07. - Complexity parameter used for Minimal Cost-Complexity Pruning=0.0. |
| 8 | 0.02 | Gradient Boosting | - Loss function to be optimized=least squares. - Learning rate shrinks the contribution of each tree by learning_rate= 0.17024124189533613 - The number of boosting stages to perform=100 - The fraction of samples to be used for fitting the individual base learners=1.0. - The function to measure the quality of a split=friedman_mse. - The minimum number of samples required to split an internal node=2 - The minimum number of samples required to be at a leaf node=14. - The minimum weighted fraction of the sum total of weights (of all the input samples) required to be at a leaf node=0.0. - Maximum depth of the individual regression estimators. The maximum depth limits the number of nodes in the tree=None. - A node will be split if this split induces a decrease of the impurity greater than or equal to this value=0.0. - An estimator object that is used to compute the initial predictions=None. - The number of features to consider when looking for the best split=None - Grow trees with max_leaf_nodes in best-first fashion=14. - Do not reuse the solution of the previous call to fit and add more estimators to the ensemble. - The proportion of training data to set aside as validation set for early stopping=0.1. - n_iter_no_change is used to decide if early stopping will be used to terminate training when validation score is not improving=4. - Tolerance for the early stopping=1e-07. - Complexity parameter used for Minimal Cost-Complexity Pruning=0.0. |

## Confounder analysis table for demographics data that are subject-specific

### Systolic Model

**Table S4 Confounder analysis table per IEEE 1708 2019a clause 4.6.2 for SBP**

| Demographic Confounder | Number of Measurements | Range | MAD (mmHg) | MADP (%) |
| --- | --- | --- | --- | --- |
| 'Age' | 72 | '20.00 - 36.00' | 4.43 | 3.60 |
|  | 88 | '36.00 - 52.00' | 6.64 | 4.87 |
|  | 71 | '52.00 - 68.00' | 5.12 | 4.16 |
|  | 55 | '68.00 - 84.00' | 8.42 (value > 7) | 5.97 |

### Diastolic Model

**Table S5 Confounder analysis table per IEEE 1708 2019a clause 4.6.2 for DBP**

| Demographic Confounder | Number of Measurements | Range | MAD (mmHg) | MADP (%) |
| --- | --- | --- | --- | --- |
| 'Age' | 72 | '20.00 - 36.00' | 3.45 | 4.55 |
|  | 88 | '36.00 - 52.00' | 4.43 | 5.01 |
|  | 71 | '52.00 - 68.00' | 3.64 | 4.98 |
|  | 55 | '68.00 - 84.00' | 4.67 | 6.34 |

# References

1 Laude, D., Weise, F., Girard, A. & Elghozi, J. L. SPECTRAL-ANALYSIS OF SYSTOLIC BLOOD-PRESSURE AND HEART-RATE OSCILLATIONS RELATED TO RESPIRATION. *Clinical and Experimental Pharmacology and Physiology* **22**, 352-357, doi:10.1111/j.1440-1681.1995.tb02014.x (1995).

2 Novak, V. *et al.* INFLUENCE OF RESPIRATION ON HEART-RATE AND BLOOD-PRESSURE FLUCTUATIONS. *Journal of Applied Physiology* **74**, 617-626, doi:10.1152/jappl.1993.74.2.617 (1993).

3 Elghozi, J. L., Laude, D. & Girard, A. EFFECTS OF RESPIRATION ON BLOOD-PRESSURE AND HEART-RATE-VARIABILITY IN HUMANS. *Clinical and Experimental Pharmacology and Physiology* **18**, 735-742, doi:10.1111/j.1440-1681.1991.tb01391.x (1991).

4 Joseph, C. N. *et al.* Slow breathing improves arterial baroreflex sensitivity and decreases blood pressure in essential hypertension. *Hypertension* **46**, 714-718, doi:10.1161/01.HYP.0000179581.68566.7d (2005).

5 Rosenberg, P. & Yancy, C. W. Noninvasive assessment of hemodynamics: an emphasis on bioimpedance cardiography. *Current Opinion in Cardiology* **15**, 151-155, doi:10.1097/00001573-200005000-00005 (2000).

6 Neto, E. P. S., Neidecker, J. & Lehot, J. J. To understand blood pressure and heart rate variability. *Annales Francaises D Anesthesie Et De Reanimation* **22**, 425-452, doi:10.1016/s0750-7658(03)00134-5 (2003).

7 Fagard, R. H., Pardaens, K. & Staessen, J. A. Relationships of heart rate and heart rate variability with conventional and ambulatory blood pressure in the population. *Journal of Hypertension* **19**, 389-397, doi:10.1097/00004872-200103000-00006 (2001).

8 Peng, R.-C. *et al.* Cuffless and Continuous Blood Pressure Estimation from the Heart Sound Signals. *Sensors (Basel)* **15**, 23653-23666, doi:10.3390/s150923653 (2015).

9 Zhang, X., MacPherson, E. & Zhang, Y. Relations Between the Timing of the Second Heart Sound and Aortic Blood Pressure. *IEEE Transactions on Biomedical Engineering* **55**, 1291-1297, doi:10.1109/TBME.2007.912422 (2008).

10 Van Der Ende, M. Y. *et al.* Causal Pathways from Blood Pressure to Larger QRS Amplitudes: a Mendelian Randomization Study. *Scientific Reports* **8**, 5817, doi:10.1038/s41598-018-24002-0 (2018).

11 Wiinberg, N. *et al.* 24-H AMBULATORY BLOOD-PRESSURE IN 352 NORMAL DANISH SUBJECTS, RELATED TO AGE AND GENDER. *Am J Hypertens* **8**, 978-986, doi:10.1016/0895-7061(95)00216-2 (1995).

12 Jaquet, F., Goldstein, I. B. & Shapiro, D. Effects of age and gender on ambulatory blood pressure and heart rate. *Journal of Human Hypertension* **12**, 253-257, doi:10.1038/sj.jhh.1000592 (1998).

13 Narkiewicz, K. *et al.* Gender-selective interaction between aging, blood pressure, and sympathetic nerve activity. *Hypertension* **45**, 522-525, doi:10.1161/01.Hyp.0000160318.46725.46 (2005).
